# IL‐15 Links Muscle–Kidney Crosstalk to Preserving Podocyte Mitochondrial Fusion and Attenuating Diabetic Nephropathy

**DOI:** 10.1002/jcsm.70256

**Published:** 2026-03-17

**Authors:** Yin Li, Jialing Rao, Weiyan Lai, Yuxiang Sun, Hongchun Lin, Jun Zhang, Zengchun Ye, Zhaoyong Hu, Hui Peng

**Affiliations:** ^1^ Nephrology Division The Third Affiliated Hospital of Sun Yat‐sen University Guangzhou China; ^2^ Nephrology Division, Department of Medicine Baylor College of Medicine Houston Texas USA

**Keywords:** diabetic nephropathy, interleukin‐15, mitochondria, optic atrophy 1, podocyte

## Abstract

**Objectives:**

High glucose induces mitochondrial dysfunction in podocytes, contributing to the development of diabetic nephropathy (DN). There is increasing evidence that muscles play a protective role by secreting myokines into the kidneys. Here, we investigated how skeletal muscle influences podocyte health via muscle–kidney crosstalk.

**Methods:**

To increase myokine release, we overexpressed PGC‐1α specifically in skeletal muscle (mPGC‐1α) and crossed these mice with db/m mice to generate diabetic mPGC‐1α:db/db mice. In parallel, db/db mice were treated intraperitoneally with recombinant murine interleukin‐15 (IL‐15). Mechanistic studies were performed using isolated primary podocytes and cultured podocyte cell lines.

**Results:**

Compared with db/db controls, mPGC‐1α:db/db mice exhibited reduced urinary albumin excretion (*p* < 0.001), mesangial matrix expansion (*p* < 0.001), glomerular basement membrane thickening (*p* < 0.001) and urinary podocin excretion (*p* < 0.001), along with increased podocyte number (*p* < 0.001). Podocytes from mPGC‐1α:db/db mice showed higher expression of Nephrin and COX IV (*p* < 0.05) and upregulation of multiple mitochondrial function‐related genes, notably OPA1 (*p* < 0.05). Skeletal muscle from mPGC‐1α:db/db mice displayed elevated IL‐15 mRNA (*p* < 0.05) and protein (*p* < 0.01) levels, accompanied by increased plasma IL‐15 concentrations (*p* < 0.05). IL‐15 treatment enhanced podocyte mitochondrial respiration, including basal oxygen consumption rate (OCR, *p* < 0.05), ATP‐coupled respiration (*p* < 0.05) and maximal respiration (*p* < 0.05). IL‐15 preserved mitochondrial fusion under high‐glucose conditions by increasing OPA1 expression (*p* < 0.05) and promoted OPA1 transcription via histone H3 acetylation at its promoter (*p* < 0.05).

**Conclusions:**

Skeletal muscle‐derived IL‐15 mediates renal protection by maintaining mitochondrial fusion in podocytes during DN progression. Targeting this pathway may offer a therapeutic strategy to preserve kidney function and slow progression to end‐stage renal disease.

## 1 Introduction

Skeletal muscle is increasingly recognized as an endocrine organ that communicates with distant tissues through the release of myokines, thereby influencing whole‐body metabolism, organ function and the progression of chronic diseases [1]. Muscle–organ crosstalk has been implicated in conditions ranging from metabolic syndrome and cardiovascular disease to chronic kidney disease (CKD), where skeletal muscle‐derived signals can confer protective effects on renal health [2]. Skeletal muscle‐specific overexpression of peroxisome proliferator‐activated receptor γ coactivator‐1α (PGC‐1α) has been shown to stimulate the secretion of myokines, such as Irisin [3] and *β*‐aminoisobutyric acid (BAIBA) [4]. Previous studies have used this transgenic mouse model to study the relationship between muscles and organs [5, 6]. In our study, we also used this model to explore whether skeletal muscle‐derived myokine influences diabetic nephropathy.

Diabetic nephropathy (DN) is a leading cause of end‐stage renal disease (ESRD) worldwide, significantly contributing to the global burden of CKD [7]. The severity of DN is closely linked to the extent of podocyte injury, which compromises the glomerular filtration barrier [8]. Podocytes, essential for maintaining glomerular filtration and integrity, are particularly vulnerable to hyperglycaemia‐induced stress. Early DN is characterized by podocyte loss, which exacerbates glomerular damage due to proteinuria and subsequent mesangial expansion [9]. The limited regenerative capacity of podocytes makes their protection critical in preventing DN progression. Pathological changes in podocytes, such as actin cytoskeleton restructuring and foot process effacement, are hallmark features of DN [10]. Consequently, safeguarding podocytes has emerged as a key therapeutic target in mitigating DN [11].

A growing body of evidence suggests that mitochondrial dysfunction, driven by high‐glucose levels, plays a pivotal role in podocyte injury and DN pathogenesis [12]. Mitochondria are highly dynamic organelles whose morphology is regulated by a balance between fission and fusion processes. These dynamics are controlled by profission proteins (e.g., Drp1 and Fis1) and profusion mediators (e.g., OPA1 and Mfn1/2), which collectively ensure mitochondrial health and function [13]. In DN, hyperglycaemia shifts this balance toward excessive fission, leading to increased production of reactive oxygen species (ROS), mitochondrial fragmentation, and ultimately podocyte apoptosis [9]. Targeting mitochondrial dynamics, particularly by restoring the balance toward fusion, is a promising strategy for mitigating podocyte injury and preventing DN progression.

Interleukin‐15 (IL‐15), which is predominantly secreted by skeletal muscle, plays roles in regulating metabolism and mitochondrial function [14]. IL‐15 has been shown to shift mitochondrial dynamics in T cells toward a fusion‐dominant state, improving mitochondrial integrity [15]. However, its impact on podocyte mitochondrial dynamics in the context of DN and the underlying mechanisms has not been explored.

Here, we investigate the role of IL‐15 in muscle–kidney crosstalk, focusing on its ability to preserve podocyte health during hyperglycaemia‐induced mitochondrial stress. Specifically, we hypothesize that IL‐15 promotes mitochondrial fusion by upregulating OPA1, counteracting high‐glucose‐induced fission and protecting podocytes from injury. By elucidating these mechanisms, our study seeks to uncover a novel therapeutic strategy for DN.

## 2 Methods

### 2.1 Cell Culture

Immortalized mouse podocytes, generously provided by Dr. Peter Mundel from Harvard University, were used for in vitro experiments. The cells were cultured in RPMI 1640 medium supplemented with 10% FBS, 1X penicillin–streptomycin (Thermo Fisher Scientific) and 20‐U/mL recombinant mouse *γ*‐IFN at 33°C to maintain a proliferative state. Differentiation was initiated by shifting the culture temperature to 37°C in the absence of *γ*‐IFN, and cells were allowed to differentiate for 10–14 days until reaching full maturity.

After differentiation, podocytes were treated with either high glucose (HG, 30 mM) or normal glucose (NG, 5.5 mM) in the presence or absence of recombinant mouse IL‐15 (1 ng/mL, R&D Systems).

Primary podocytes were isolated from mouse kidneys using a modified magnetic bead‐based protocol [16]. Briefly, mice were anaesthetised and perfused via the left ventricle with PBS containing Dynabeads (4 × 10^7^ beads/mL) to selectively label glomeruli. Kidneys were excised, and cortical tissues were carefully dissected and minced. The tissue fragments were digested in Collagenase I (2.5 mg/mL in HBSS) at 37°C for 30 min with gentle shaking to liberate intact glomeruli. The digested suspension was passed through a 150‐μm Pluriselect pluriStrainer (Fisher Scientific) to remove large debris, and the filtrate was placed on a magnetic stand to collect bead‐labelled glomeruli. The glomerular pellet was washed three times with cold PBS. To release podocytes, the purified glomeruli were then digested with 0.05% trypsin–EDTA at 37°C for 15 min. The digestion was stopped with serum‐containing medium, and the suspension was filtered through a 40‐μm cell strainer to remove residual glomerular fragments. The flow‐through containing dissociated podocytes was centrifuged and resuspended in RPMI 1640 medium for culture and downstream assays.

### 2.2 Animal Experiments

All animal procedures were approved by the Institutional Animal Care and Use Committee at Baylor College of Medicine. Muscle‐specific PGC‐1α transgenic mice (C57BL/6 J background; Jackson Laboratory, Bar Harbour, ME) were crossed with C57BLKS‐Lepr^db/m^ mice for at least three generations to generate four experimental groups: db/m, db/db, mPGC‐1α:db/m and mPGC‐1α:db/db.

For IL‐15 treatment, the dose and injection schedule were selected based on preliminary efficacy testing and prior literature aiming to model physiologically relevant IL‐15 signalling rather than pharmacological immune activation. Low‐dose IL‐15 has been reported to exert metabolic and muscle‐related effects without inducing overt immune stimulation and is substantially lower than doses typically used in oncologic or immunotherapy settings [17]. In preliminary experiments, daily IL‐15 administration resulted in mild renal interstitial inflammation, whereas alternate‐day dosing preserved efficacy with reduced adverse effects. Accordingly, mice received intraperitoneal injections of recombinant murine IL‐15 (20 μg/kg; PeproTech, Cat#210‐15) every other day for 4 weeks. Control mice received an equivalent volume of PBS. Body weight and fasting glucose were monitored weekly. At study completion, mice were anaesthetised, euthanized and kidneys were collected for histological and molecular analyses.

### 2.3 Mitochondrial Respiration Assay

Differentiated podocytes were seeded in 24‐well XF24 Cell Culture Microplates (Seahorse Bioscience) at a density of 5 × 10^3^ cells per well. One day prior to the assay, the sensor cartridge was hydrated in Seahorse XF Calibrant overnight in a non‐CO_2_ incubator. The Seahorse XF Cell Mito Stress Test was performed according to the manufacturer's protocol, with sequential injections of oligomycin (1 μM), FCCP (1 μM) and rotenone/antimycin A (0.5 μM each). Data were analysed using Seahorse Wave software (Agilent), and oxygen consumption rate (OCR) values were normalized to protein content per well.

### 2.4 Single‐Cell Data Analysis

scRNA‐seq data of human and mice diabetic kidneys were obtained from the GEO dataset (GSE131882 and GSE184652, respectively). We applied Seurat v4 for downstream analyses, including normalization, scaling and clustering of cells. Batch effects were corrected by using harmony R package (version 1.2.1), and quality control strategy was performed according to the original article [18]. R package limma (version 3.61.12) was used for different analyses on podocytes, while we inputted the results for further GO enrichment analysis by using clusterProfiler R package (version 4.13.4).

### 2.5 Statistical Analysis

All data were analysed using GraphPad Prism 9 (GraphPad Software, San Diego, CA). Results are presented as mean ± standard error of the mean (SEM). Comparisons between two groups were performed using unpaired two‐tailed Student's *t*‐tests. For comparisons involving multiple groups, two‐way analysis of variance (ANOVA) was used with appropriate post hoc tests (Tukey's or Bonferroni's) to determine group differences. Statistical significance was defined as *p* < 0.05. Sample sizes (biological replicates) for each experiment are reported in the corresponding figure legends.

## 3 Results

### 3.1 Muscle‐Specific PGC‐1α Alleviates Podocyte Injury in Mice With DN

**FIGURE 1:**
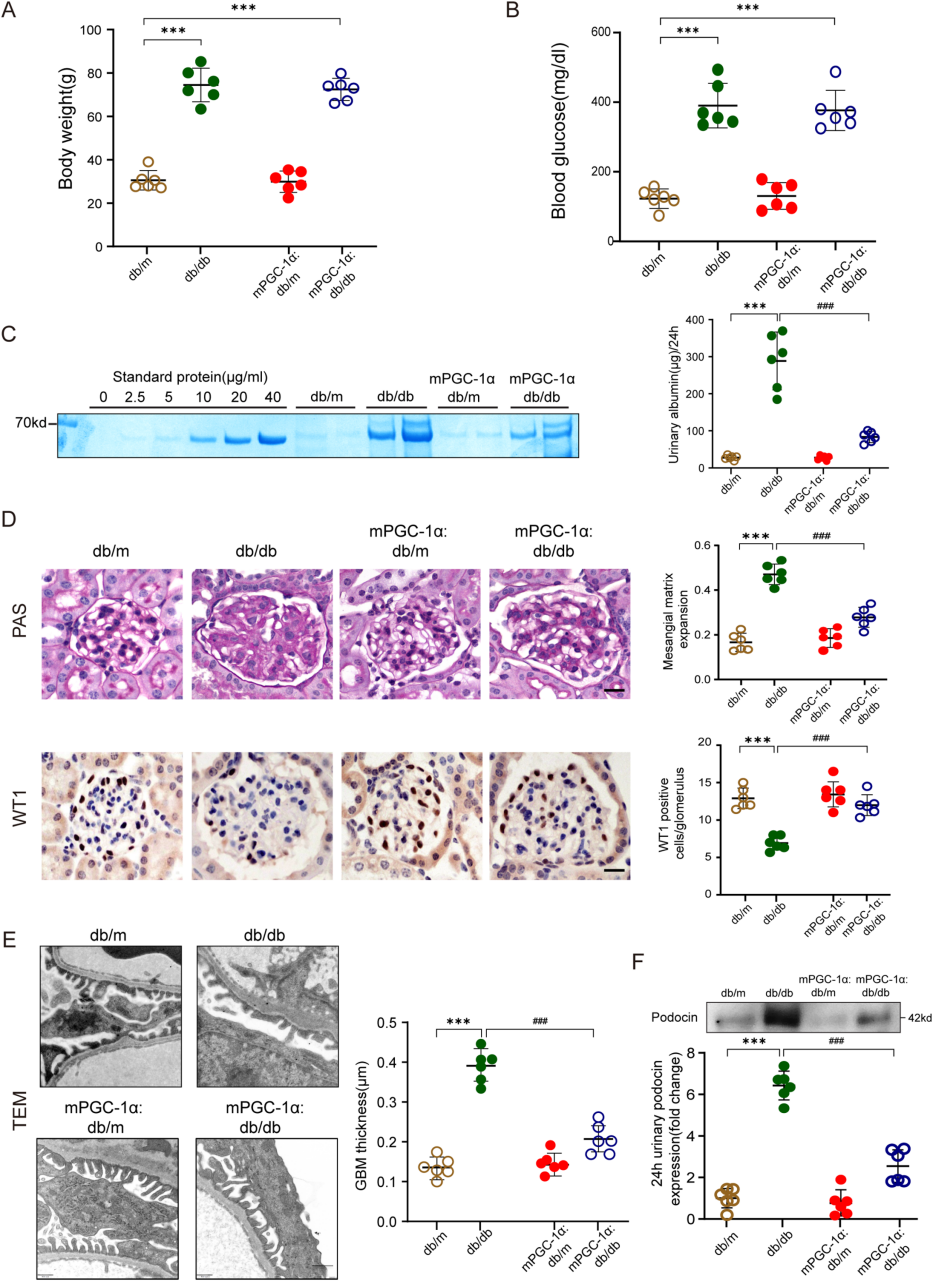
Overexpression of PGC‐1α in skeletal muscles ameliorates DN in mice. (A) Body weight measurements of 28‐week‐old mice in each experimental group. Data are presented as mean ± SEM (***p < 0.001 vs. db/m mice; two‐way ANOVA; *n* = 6 per group). (B) Blood glucose levels of 28‐week‐old mice in each group. Data are presented as mean ± SEM. (***p < 0.001 vs. db/m mice; two‐way ANOVA; *n* = 6 per group). (C) Left panel: Representative Coomassie‐stained SDS‐PAGE gel showing 24‐h urinary albumin precipitation. Right panel: Quantitative analysis of urinary albumin excretion over 24 h. Data are presented as mean ± SEM. (***p < 0.001 vs. db/m mice; ###*p* < 0.001 vs. db/db mice; two‐way ANOVA; *n* = 6 per group). (D) Left upper panel: Representative PAS‐stained kidney sections highlighting mesangial expansion. Right upper panel: Quantitative analysis of mesangial matrix expansion. Left lower panel: Representative WT1 immunostaining of podocytes in kidney sections. Right lower panel: Quantification of WT1‐positive podocytes per glomerulus, calculated from 20 randomly selected glomeruli per animal. Scale bar: 20 μm. Data are presented as mean ± SEM. (***p < 0.001 vs. db/m mice; ###*p* < 0.001 vs. db/db mice; two‐way ANOVA; *n* = 6 per group). (E) Left panel shows representative transmission electron micrographs (TEM) of the glomerular filtration barrier showing podocyte foot processes and glomerular basement membrane (GBM) thickness. Scale bar: 500 nm. Right panel shows quantification of GBM thickness based on TEM analysis. Data are presented as mean ± SEM. (***p < 0.001 vs. db/m mice; ###*p* < 0.001 vs. db/db mice; two‐way ANOVA; *n* = 6 per group). (F) Top: Representative Western blot of podocin levels in 24‐h urinary precipitates. Bottom: Quantitative analysis of podocin protein expression normalized to loading control. Data are presented as mean ± SEM. (***p < 0.001 vs. db/m mice; ###*p* < 0.001 vs. db/db mice; two‐way ANOVA; *n* = 6 per group).

To investigate the role of muscle–kidney crosstalk in diabetes‐induced kidney injury, we developed a diabetic mouse model with muscle‐specific PGC‐1*α* overexpression (mPGC‐1α). By crossing db/m mice with mPGC‐1α mice, we generated four experimental groups: db/m, db/db, mPGC‐1α:db/m, and mPGC‐1α:db/db. At 28 weeks of age, there were no significant differences in body weight (Figure 1A) or blood glucose levels (Figure 1B) between mPGC‐1α:db/db and db/db mice, indicating that the observed effects of PGC‐1α overexpression were not due to systemic metabolic alterations. However, mPGC‐1α:db/db mice exhibited a significant reduction in 24‐h urinary albumin excretion compared to db/db mice (Figure 1C), highlighting improved renal function. Consistent with reduced albuminuria, histological analysis of kidney sections stained with periodic acid‐Schiff (PAS) demonstrated that mPGC‐1α:db/db mice showed significantly decreased mesangial expansion and glomerulosclerosis compared to db/db mice (Figure 1D). These findings suggest that muscle‐specific PGC‐1α expression mitigates the development of DN in mice. Given the pivotal role of podocyte injury in DN progression, we next examined podocyte health. Immunostaining for WT1, a podocyte‐specific marker, revealed a significant decrease in WT1‐positive podocytes in the glomeruli of db/db mice compared to db/m controls. Notably, this reduction was significantly ameliorated in mPGC‐1α:db/db mice (Figure 1D), indicating a protective effect of muscle‐specific PGC‐1α expression on podocyte number. To further evaluate podocyte morphology, transmission electron microscopy (TEM) was performed. TEM analysis showed that mPGC‐1α:db/db mice exhibited reduced foot process effacement and decreased glomerular basement membrane (GBM) thickening compared to db/db mice (Figures 1E). These observations suggest preserved podocyte structure in mPGC‐1α:db/db mice. Finally, we measured urinary podocin levels as a marker of podocyte injury. Diabetes‐induced increases in urinary podocin levels were significantly reduced in mPGC‐1α:db/db mice compared to db/db controls (Figure 1F). This further supports the role of muscle‐specific PGC‐1α in protecting against podocyte injury and preserving glomerular integrity. Together, these findings demonstrate that mPGC‐1α overexpression alleviates podocyte injury and morphological abnormalities in DN, providing evidence for the protective role of muscle‐specific PGC‐1α in kidney health.

### 3.2 mPGC‐1α Prevents Mitochondrial Injury in Podocytes of Diabetic Mice

**FIGURE 2:**
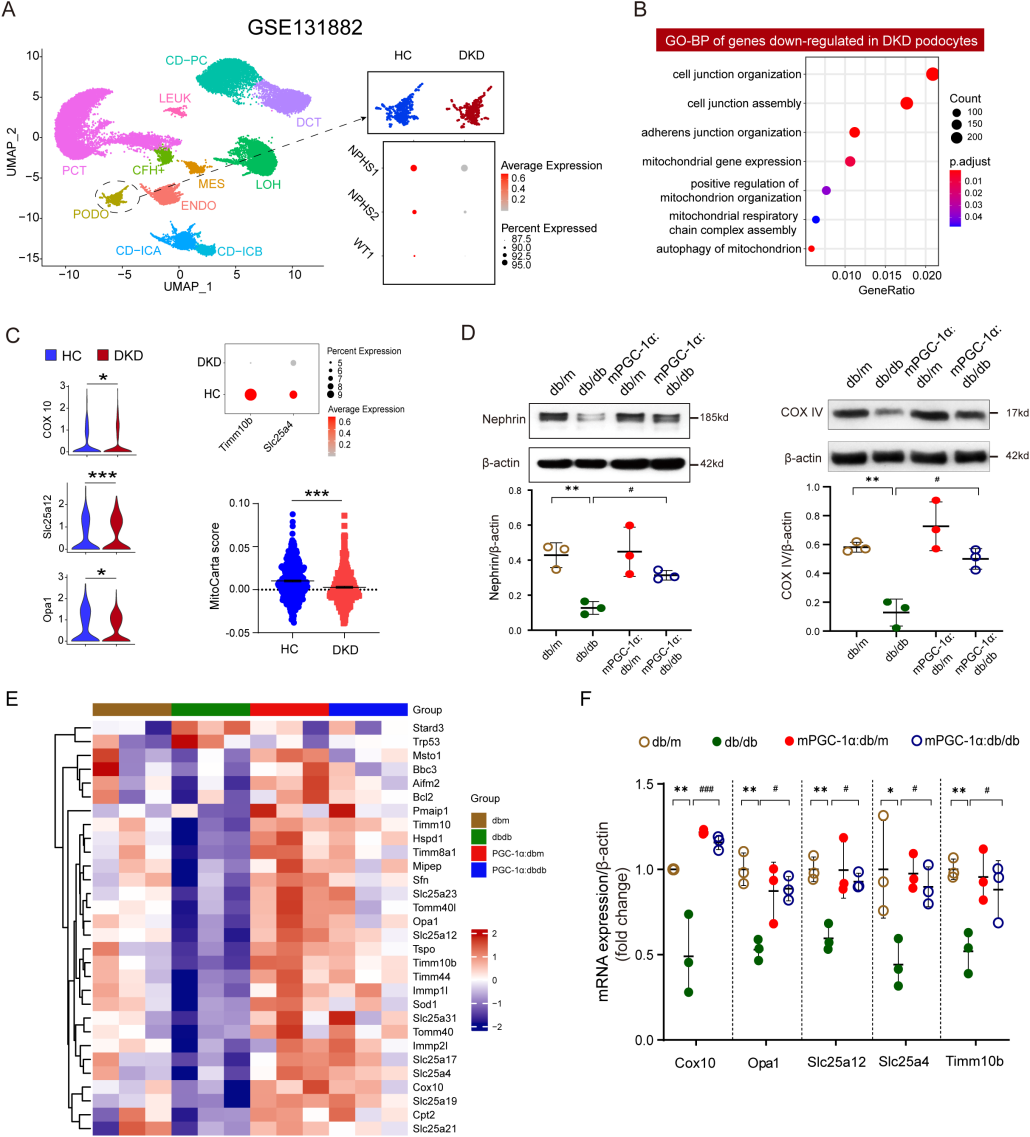
mPGC‐1α prevents podocytes mitochondria damage in diabetic mice. (A) Uniform Manifold Approximation and Projection (UMAP) plot from scRNA‐seq data showing 11 distinct kidney cell clusters in healthy control (HC) and diabetic kidney disease (DKD) samples. Identified cell types include collecting duct‐principal cell (CD‐PC), distal convoluted tubule (DCT), leukocyte (LEUK), loop of Henle (LOH), mesangial cell (MES), complement factor H‐positive cells (CFH+), proximal convoluted tubule (PCT), podocytes (PODO), endothelial cells (ENDO), collecting duct‐intercalated cell A (CD‐ICA) and collecting duct‐intercalated cell B (CD‐ICB). (B) Dot plots displaying Gene Ontology (GO) enrichment analysis of downregulated genes in podocytes from DKD samples, with a focus on pathways related to mitochondrial dysfunction. (C) Violin plots illustrating the expression levels of mitochondrial function‐related genes (e.g., *Cox10*, *Opa1* and *Slc25a12*) in podocytes. *Y*‐axis represents log2‐normalized read counts. Expression levels of *Timm10b* and *Slc25a4* in podocytes of healthy control (HC) and diabetic kidney disease (DKD) group are shown in a bubble chart. MitoCarta score showing reduced mitochondrial transcriptional activity in DKD podocytes compared with HC. (D) Representative Western blot images (left upper panel) of Nephrin protein expression in podocytes from db/m, db/db, mPGC‐1α:db/m and mPGC‐1α:db/db mice. Bar graphs (left lower panel) quantify Nephrin expression levels. Representative Western blot images (right upper panel) of COX IV protein expression in podocytes from each group. Quantitative analysis (right lower panel) of COX IV expression is shown. Data are presented as mean ± SEM (**p < 0.01 vs. db/m; #*p* < 0.05 vs. db/db by two‐way ANOVA; *n* = 3 per group). (E) Heat map showing relative expressions (*Z*‐score normalized) of the 30 changed mitochondria genes in podocytes from four groups of mice: db/m (n = 3), db/db (*n* = 3), PCG‐1α: db/m (*n* = 3), PCG‐1α: db/db (*n* = 3). (F) RT‐qPCR validation of mRNA expression levels of mitochondrial biogenesis‐related genes (*Opa1*, *Cox10*, *Slc25a12*, *Slc25a4 and Timm10b*) in podocytes from each group. Downregulated expression in db/db mice was significantly restored in mPGC‐1α:db/db mice. Data are presented as mean ± SEM (*p < 0.05 or *p < 0.01 vs. db/m; #*p* < 0.05 or ###*p* < 0.001 vs. db/db by two‐way ANOVA; *n* = 3 per group).

To investigate the mechanisms underlying podocyte injury in DN, we analysed scRNA‐seq data from kidney samples of three healthy controls (HC) and three DKD patients from a previous study (GSE131882). Following established cell type annotation methods, we identified 11 kidney cell types, including podocytes, and observed significant downregulation of nephrin (NPHS1 and NPHS2) and WT1, markers of podocyte integrity, in the DKD group compared to healthy controls (Figure 2A). Gene Ontology (GO) analysis further revealed enriched pathways associated with mitochondrial dysfunction in DKD podocytes, highlighting mitochondrial damage as a potential contributor to podocyte injury (Figure 2B). Consistent with these findings, transcription levels of key mitochondrial genes, including *Cox10*, *Opa1*, *Slc25a12*, *Slc25a4 and Timm10b*, were significantly reduced in DKD podocytes (Figure 2C). To systematically assess mitochondrial transcriptional status, we used the Human MitoCarta 3.0 reference set and calculated a podocyte mitochondrial module score using Seurat's AddModuleScore. This analysis demonstrates a significant reduction in mitochondrial gene programme activity in DKD podocytes (Figure 2C).

To explore the protective effects of mPGC‐1α on podocytes, we isolated primary podocytes from db/m, db/db, mPGC‐1*α*:db/m and mPGC‐1*α*:db/db mice. Nephrin, a key slit diaphragm protein and marker of podocyte integrity, which was markedly reduced in db/db mice, was significantly restored in mPGC‐1α:db/db mice (Figure 2D). Similarly, Cytochrome c Oxidase Subunit IV (COX IV), a nuclear‐encoded component of the mitochondrial electron transport chain (ETC), showed significantly improved expression in podocytes of mPGC‐1α:db/db mice compared to db/db controls (Figure 2D). These findings suggest that mPGC‐1α protects podocytes by preserving both structural integrity and mitochondrial functionality. Given that mitochondrial dysfunction is an early event in podocyte injury during diabetes, we next examined mitochondrial gene expression profiles using a qPCR‐based mRNA array targeting mitochondrial biogenesis and function. Out of 84 tested genes, 30 differentially expressed genes are shown as a heat map. Among them, 28 genes were significantly downregulated in the podocytes of db/db mice compared to db/m controls. Notably, mPGC‐1α overexpression in mPGC‐1α:db/db mice prevented the downregulation of these genes, restoring their expression to levels comparable to db/m mice (Figures 2E). Among the preserved genes was Optic Atrophy 1 (*Opa1*), a critical regulator of mitochondrial inner membrane fusion and biogenesis. To validate these findings, we performed real‐time PCR on key mitochondrial biogenesis genes, including *Opa1*, *Cox10*, *Slc25a12*, *Slc25a4 and Timm10b*. All these genes showed significantly improved expression in podocytes of mPGC‐1α:db/db mice compared to db/db controls, further confirming the protective role of mPGC‐1α in maintaining mitochondrial gene expression (Figure 2F). In summary, these results demonstrate that mPGC‐1α overexpression prevents diabetes‐induced mitochondrial dysfunction in podocytes by restoring the expression of key genes involved in mitochondrial biogenesis and function. These findings highlight the protective effects of mPGC‐1α against mitochondrial injury as a mechanism for alleviating DN.

### 3.3 Plasma From mPGC‐1α Mice Prevents High‐Glucose‐Induced Injury and Mitochondrial Dysfunction in Podocyte

**FIGURE 3:**
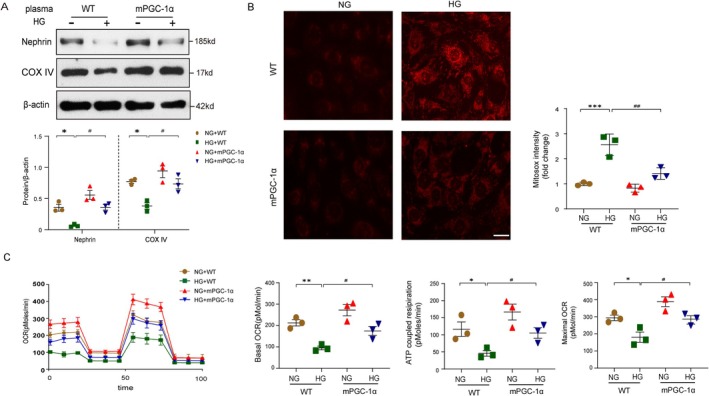
Plasma from mPGC‐1α mice prevents podocytes injury induced by high glucose. (A) Representative Western blot images (upper panel) showing Nephrin and COXIV expression in podocytes incubated with plasma from wild type (WT) or plasma from mPGC‐1α mice, followed by high glucose (HG, 30 mM) or normal glucose (NG, 5.5 mM) treatment. Bar graphs (lower panel) quantify Nephrin and COXIV expression levels. Data are presented as mean ± SEM (*p < 0.05 vs. WT plasma under NG; #*p* < 0.05 vs. WT plasma under HG; two‐way ANOVA; *n* = 3 per group). (B) Representative immunofluorescence images display mitochondrial reactive oxygen species (ROS) in mouse podocytes, stained with MitoSOX Red. Scale bars indicate 50 μm. Bar graphs present the quantitative analysis of mitochondrial ROS in podocytes. (***p < 0.001 vs. podocytes with WT plasma under NG; ##*p* < 0.01 vs. podocytes with WT plasma under HG; two‐way ANOVA, *n* = 3 per group). C. Cellular respiration in podocytes was assessed using a Mito Stress Assay, with sequential additions of oligomycin, carbonyl cyanide 4‐(trifluoromethoxy) phenylhydrazone (FCCP) and antimycin. Bar graphs display statistical analyses of basal OCR, ATP‐coupled respiration and maximal respiration. (*p < 0.05 or **p < 0.01 vs. WT plasma under NG; #*p* < 0.05 vs. WT plasma under HG; two‐way ANOVA, *n* = 3 per group).

Given that muscle‐specific PGC‐1α overexpression protects against diabetes‐induced mitochondrial injury in podocytes, we investigated whether plasma from mPGC‐1α mice contains factors that mediate this protective effect. Cultured podocytes were incubated with plasma from wild type (WT) or mPGC‐1α mice for 2 h, followed by high‐glucose (HG, 30 mM) treatment for 72 h. Both Nephrin and COXIV were significantly upregulated in podocytes treated with mPGC‐1α plasma compared to WT plasma after HG exposure (Figure 3A). High glucose is known to induce excessive mitochondrial ROS production, leading to podocyte injury. Using MitoSOX staining, we observed that plasma from mPGC‐1α mice significantly suppressed HG‐induced ROS overproduction in podocytes (Figures 3B). To further evaluate mitochondrial function in podocytes, we measured oxygen consumption rate (OCR) using a Seahorse X24 Extracellular Flux Analyser. In the presence of HG, treatment with WT plasma significantly reduced basal respiration, ATP‐linked OCR and maximal respiratory capacity, indicating mitochondrial dysfunction. In contrast, plasma derived from mPGC‐1α transgenic mice effectively restored all of them in HG‐treated podocytes, suggesting improved mitochondrial respiratory capacity (Figures 3C). Collectively, these findings indicate that plasma from mPGC‐1α mice harbours circulating factors, possibly myokines, that protect podocytes from HG‐induced mitochondrial dysfunction. These results underscore the systemic protective effects of muscle‐specific PGC‐1α overexpression in the context of diabetic nephropathy.

### 3.4 Identification of the Myokine That Protects Against HG‐Induced Mitochondrial Dysfunction in Podocytes

**FIGURE 4:**
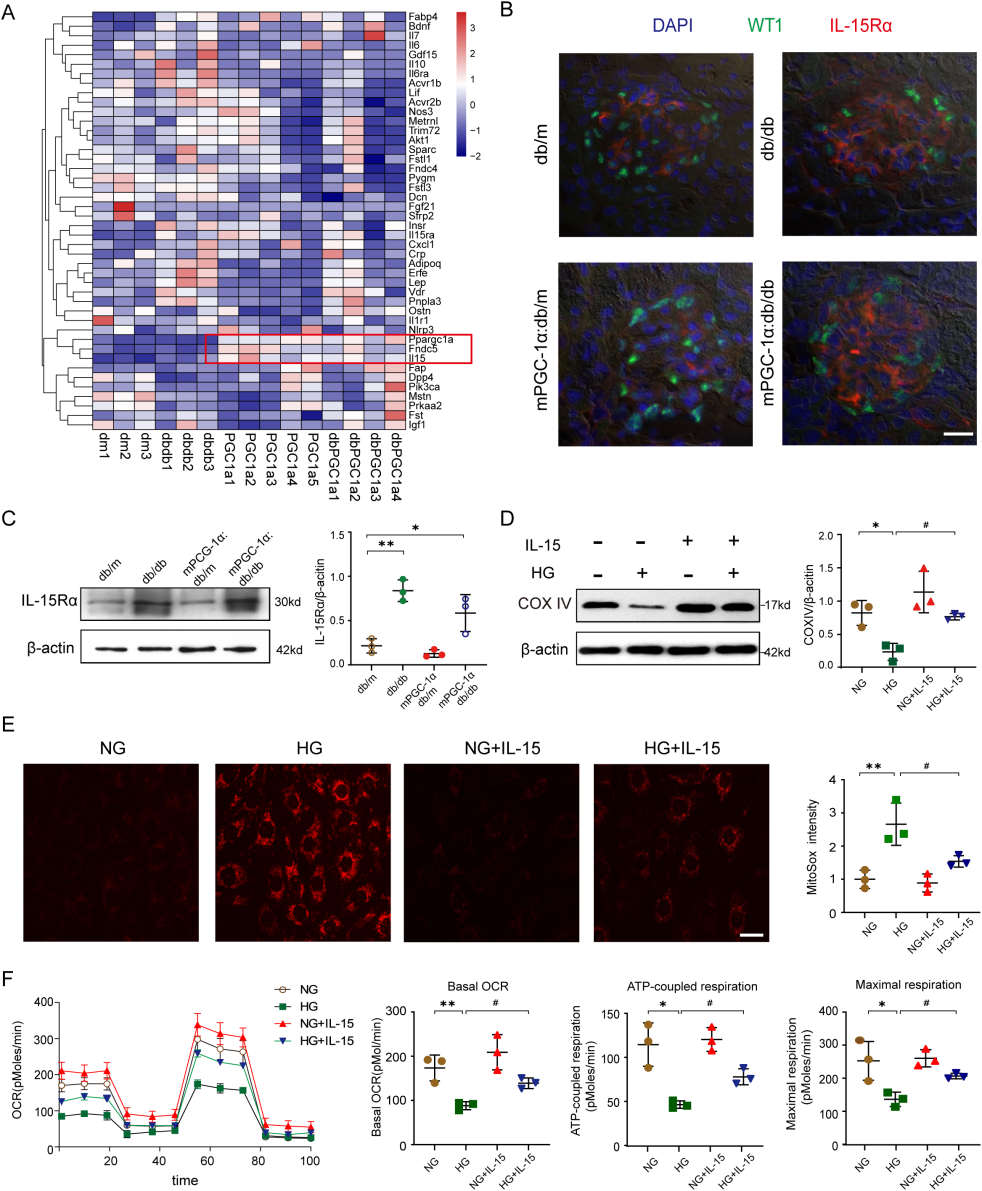
IL‐15 protects against HG‐induced mitochondria dysfunction in podocytes. (A) Heat map showing relative expressions of the 44 myokines in muscles from four groups of mice: db/m (*n* = 3), db/db (*n* = 3), PCG1α: db/m (*n* = 5), PCG1α: db/db (*n* = 4). The red box marks the top3 myokines with significant changes. (B) Confocal microscopy images with double immunofluorescence staining depict the expression of IL‐15Rα (red) and WT1 (green) in kidney sections from each mouse group. Cell nuclei are stained with DAPI (blue). Scale bars represent 20 μm. (C) Representative Western blots (left panel) display IL‐15Rα expression in podocytes from each mouse group. The bar graph (right panel) quantifies this expression. (*p < 0.05 or **p < 0.01 vs. db/m; two‐way ANOVA, *n* = 3 per group). (D) Representative Western blots (left panel) show COX IV expression in podocytes, with quantitative analysis provided in the right panel. (*p < 0.05 vs. NG; #*p* < 0.05 vs. HG; two‐way ANOVA, *n* = 3 per group). (E) Representative immunofluorescence images illustrate mitochondrial reactive oxygen species (ROS) in podocytes stained with MitoSOX Red. The bar graph quantifies mitochondrial ROS levels, assessed by fluorescent intensity. Scale bars indicate 50 μm. (**p < 0.01 vs. NG; #*p* < 0.05 vs. HG; two‐way ANOVA, *n* = 3 per group). F. Cellular respiration in podocytes was evaluated using a Mito Stress Assay, featuring sequential additions of oligomycin, carbonyl cyanide 4‐(trifluoromethoxy) phenylhydrazone (FCCP) and antimycin. The bar graph displays statistical analyses of basal OCR, ATP‐coupled respiration and maximal respiration. (*p < 0.05 or **p < 0.01 vs. NG; #*p* < 0.05 vs. HG; two‐way ANOVA, *n* = 3 per group).

Since mPGC‐1α overexpression in muscles is known to stimulate myokine secretion, we hypothesized that specific myokines in plasma might mediate podocyte protection and prevent mitochondrial dysfunction under high‐glucose (HG) conditions. To identify these myokines, we performed transcriptome sequencing of muscles from four mouse groups: db/m, db/db, mPGC‐1α:db/m and mPGC‐1α:db/db. Gene enrichment analysis highlighted the upregulation of metabolic and respiratory pathways in mPGC‐1α mice, consistent with previous findings (Figure S1A,B). Among the identified myokines, *Ppargc1a (Pgc‐1α)*, *Fndc5 (Irisin)* and *Il‐15* were consistently upregulated in mPGC‐1α:db/m and mPGC‐1α:db/db mice compared to controls (Figure 4A). We further validated the expression of seven myokines associated with mitochondrial function (e.g., *Bdnf*, *Ctsb*, *Fgf‐21* and *Il‐15*) in muscle tissue using qPCR. Among these, *Il‐15* showed the most robust changes, with significant upregulation in mPGC‐1α:db/m mice and preservation in mPGC‐1α:db/db mice, whereas *Il‐15* expression was reduced in db/db mice (Figure S1C). We have also performed proteomic analysis of skeletal muscle from mPGC‐1α and control mice, which independently confirmed that IL‐15 protein levels are significantly upregulated in mPGC‐1α mice, with or without diabetes (Figure S1D,E). Plasma IL‐15 concentrations, measured by ELISA, mirrored the mRNA expression trends, confirming systemic upregulation of IL‐15 in mPGC‐1α mice (Figure S1F). To explore whether IL‐15 could affect podocytes, we examined the expression of IL‐15 receptor alpha (IL‐15Rα) in podocytes. Double immunofluorescence staining for IL‐15Rα and WT1 (a podocyte marker) showed minimal IL‐15Rα expression in glomeruli from db/m and mPGC‐1α:db/m mice. In contrast, IL‐15Rα expression was significantly increased in glomeruli from db/db and mPGC‐1α:db/db mice, with colocalization in WT1‐positive podocytes (Figure 4B). Immunoblotting confirmed the presence of IL‐15Rα in podocytes isolated from diabetic mice, indicating that diabetes induces IL‐15Rα expression, potentially enabling IL‐15 to act on podocytes (Figure 4C). We also performed PCR analysis of *Il‐15rα* in cultured podocytes stimulated under four conditions: normal glucose (NG), high glucose (HG), NG + IL‐15 and HG + IL‐15. The results demonstrated that HG significantly increased *Il‐15rα* expression (Figure S2A), showing a pattern consistent with our in vivo observations. Moreover, we analysed *Il‐15rα* expression in podocytes from publicly available single‐cell sequencing data (GSE184652). Similarly, we found elevated expression of *Il‐15rα* in podocytes of diabetic mice compared to control mice (Figure S2B). These complementary approaches strengthen our conclusion about the glucose‐dependent regulation of IL‐15Rα in podocytes.

To confirm IL‐15's functional role, we tested its effects on HG‐induced mitochondrial dysfunction in podocytes. Treatment with IL‐15 restored COX IV levels in HG‐treated podocytes (Figure 4D) and significantly reduced mitochondrial ROS production, as shown by MitoSOX staining (Figure 4E). Furthermore, Seahorse assay analysis revealed that IL‐15 improved key mitochondrial respiration parameters, including basal oxygen consumption rate (OCR), ATP‐coupled OCR and maximal OCR, which were significantly impaired by HG (Figure 4F). These effects closely mirrored the protective effects observed with plasma from mPGC‐1α mice. In summary, our findings identify IL‐15 as a key myokine secreted by mPGC‐1α‐overexpressing muscles. Diabetes‐induced IL‐15Rα expression in podocytes enables IL‐15 to mitigate HG‐induced mitochondrial dysfunction, improving mitochondrial respiration and reducing ROS production.

### 3.5 IL‐15 Counteracts HG‐Induced Mitochondrial Fission in Podocytes

**FIGURE 5:**
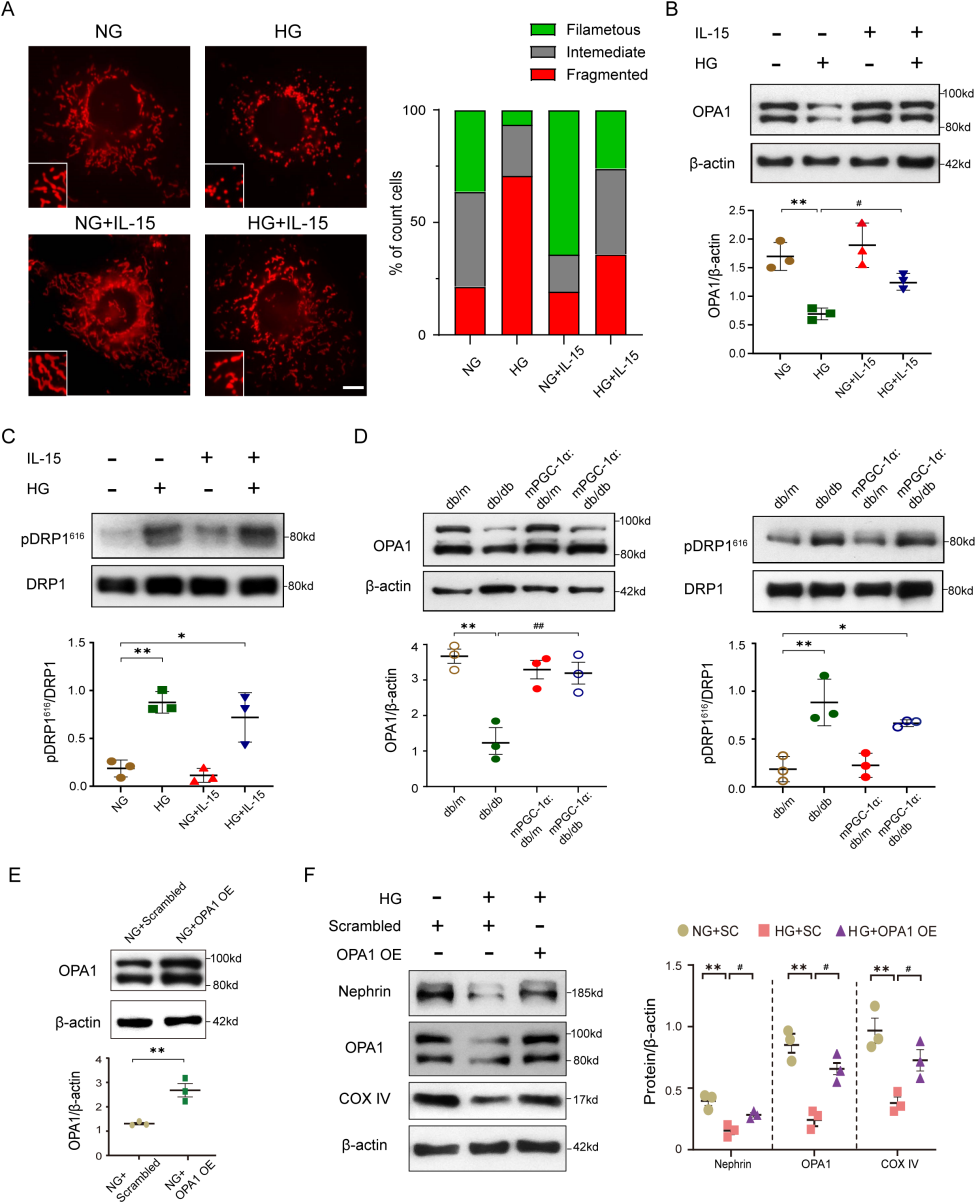
IL‐15 preserves mitochondrial fusion against high‐glucose injury in podocyte via increasing OPA1 expression. (A) Representative immunofluorescence images illustrate mitochondrial morphology in cultured podocytes using MitoTracker Red staining and quantification of mitochondrial morphologies from the indicated groups. Scale bars represent 10 μm. (B) Upper panel shows representative Western blots of OPA1 expression in cultured podocytes. Quantitative analysis is provided in the lower panel. The quantitative analysis of OPA1 is calculated with total OPA1 levels. (**p < 0.01 vs. NG; #*p* < 0.05 vs. HG; two‐way ANOVA, *n* = 3 per group). (C) Upper panel shows representative Western blots of DRP1 phosphorylation levels at serine 616 expression in cultured podocytes. Quantitative analysis is provided in the lower panel. (*p < 0.05 or **p < 0.01 vs. NG; two‐way ANOVA, *n* = 3 per group). (D) Upper panel presents representative Western blots of OPA1 and DRP1 phosphorylation levels at serine 616 expression in podocytes from db/m, db/db, mPGC‐1α:db/m and mPGC‐1α:db/db mice. Quantitative analysis is in the lower panel. (*p < 0.05 or **p < 0.01 vs. db/m; ##*p* < 0.01 vs. db/db; two‐way ANOVA, *n* = 3 per group). (E) Upper panel shows representative Western blots of OPA1 expression in cultured podocytes transfected with or without OPA1 overexpression adenovirus. Quantitative analysis is provided in the lower panel. (**p < 0.01 vs. NG + Scrambled; *t*‐test, *n* = 3 per group). (F) Left panel shows representative Western blots of Nephrin, OPA1 and COX IV in cultured podocytes subjected to HG stimulation and OPA1 overexpression. Quantitative analysis of protein expression is on the right panel. (SC: Scrambled; **p < 0.01 vs. NG + scrambled; #*p* < 0.05 vs. HG + scrambled; two‐way ANOVA, *n* = 3 per group).

To investigate the mechanism by which IL‐15 protects podocytes, we examined morphological changes in mitochondria using MitoTracker staining. High‐glucose (HG) treatment resulted in a marked increase in small, punctate mitochondria, indicative of mitochondrial fission, compared to the filamentous mitochondrial network observed under normal glucose (NG) conditions. Preincubation with IL‐15 prevented this HG‐induced mitochondrial fission, maintaining a more filamentous mitochondrial morphology (Figure 5A). It suggests that IL‐15 effectively counteracts HG‐induced mitochondrial fission in podocytes. Next, we investigated the expression of key regulators of mitochondrial dynamics: Optic Atrophy 1 (OPA1), a protein essential for mitochondrial fusion, and Dynamin‐Related Protein‐1 (DRP1), a major regulator of mitochondrial fission. Western blot analysis revealed that IL‐15 significantly upregulated OPA1 expression in podocytes treated with HG (Figure 5B). Concurrently, HG treatment increased DRP1 phosphorylation, indicative of enhanced mitochondrial fission. However, IL‐15 preincubation did not significantly alter DRP1 phosphorylation levels (Figure 5C). These findings suggest that IL‐15 selectively promotes mitochondrial fusion without directly modulating mitochondrial fission. We further examined OPA1 and DRP1 expression in primary podocytes isolated from our mouse models. Immunoblotting confirmed that OPA1 expression was increased in mPGC‐1α:db/db mice compared to db/db mice, consistent with in vitro observations. However, DRP1 phosphorylation levels were similar between mPGC‐1α:db/db and db/db mice, aligning with the findings that IL‐15 does not directly affect DRP1‐mediated fission (Figure 5D). To elucidate the role of OPA1 in high‐glucose‐induced mitochondrial dysfunction in podocytes, we transfected cells with an OPA1‐overexpressing adenovirus and confirmed successful overexpression by Western blotting (Figure 5E). Moreover, under high‐glucose conditions, OPA1 overexpression increased the protein levels of both Nephrin and COX IV compared to the HG + scrambled group (Figure 5F). These data suggest that high‐glucose‐induced podocyte injury is partly mediated by OPA1 downregulation. In summary, our results demonstrate that IL‐15 protects podocytes from HG‐induced mitochondrial dysfunction by enhancing mitochondrial fusion through OPA1 upregulation. This suggests that IL‐15 acts as a key regulator of mitochondrial dynamics, counteracting the harmful effects of HG‐induced mitochondrial fission.

### 3.6 OPA1 Is Required for IL‐15 Maintaining Fused Mitochondria Network in Podocytes

**FIGURE 6:**
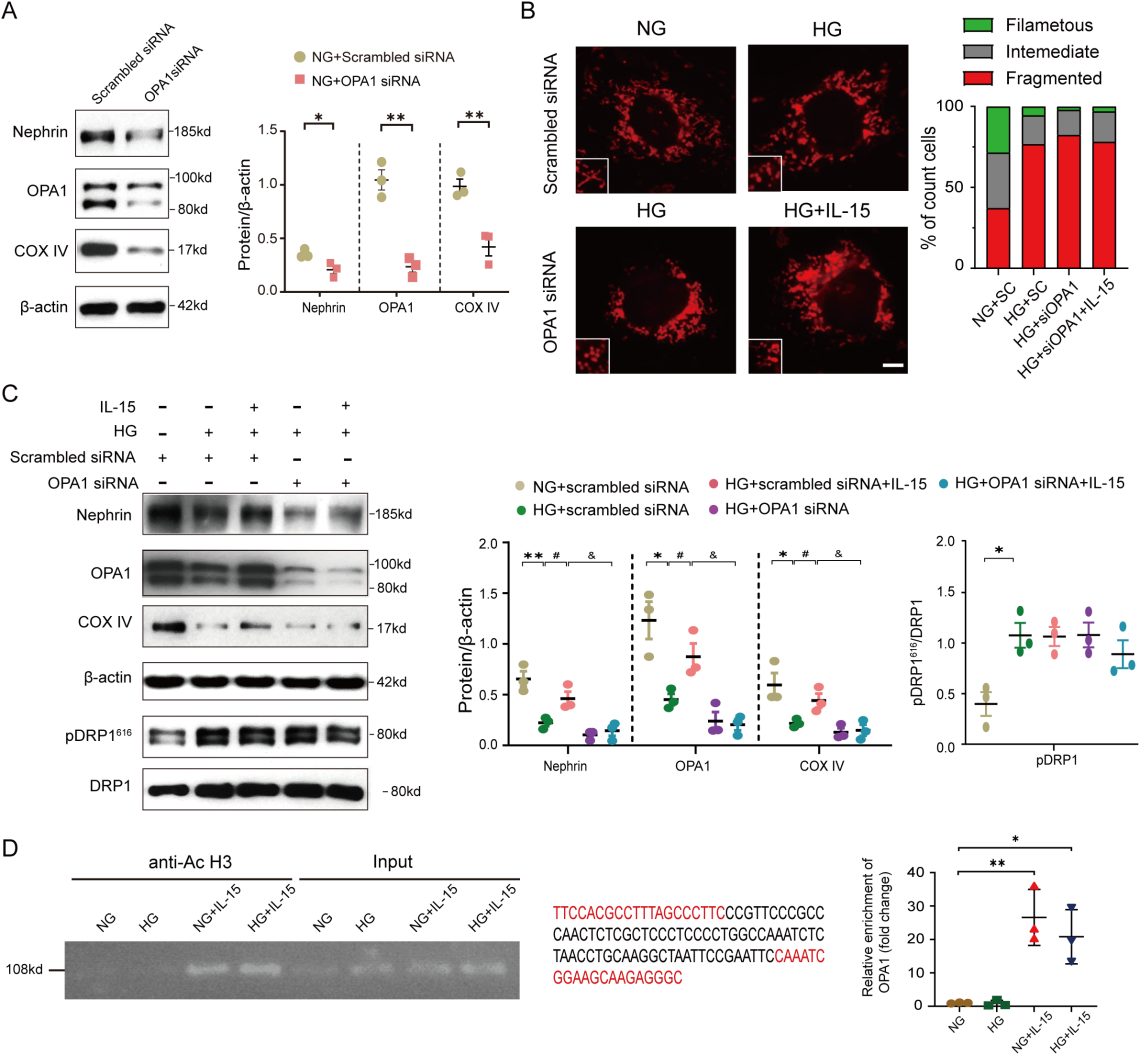
OPA1 is required for IL‐15‐mediated protection against HG‐induced podocytes injury. (A) Representative Western blots confirm OPA1 knockdown in podocytes transfected with OPA1 siRNA or scrambled siRNA (control) for 48 h. Quantitative analysis is in the right panel. (*p < 0.05 or **p < 0.01 vs. podocytes transfected with scrambled siRNA; *t*‐test, *n* = 3 per group). (B) Representative immunofluorescence images display mitochondrial morphology in podocytes stained with MitoTracker Red and quantification of mitochondrial morphologies from the indicated groups. Scale bars indicate 10 μm. (C) Left panel shows representative Western blots of Nephrin, OPA1, COX IV and DRP1 phosphorylation levels at serine 616 in cultured podocytes subjected to HG stimulation and OPA1 knockdown, followed by IL‐15 treatment. Quantitative analysis of protein expression are provided in the right panel. (*p < 0.05 or **p < 0.01 vs. NG + scrambled siRNA; #*p* < 0.05 vs. HG + scrambled siRNA; &*p* < 0.05 vs. HG + scrambled siRNA + IL‐15; two‐way ANOVA, *n* = 3 per group). (D) Left panel presents PCR analyses of DNA products from immunoprecipitation with antiacetyl‐histone H3 (anti‐H3ac) in podocytes. Middle panel shows the OPA1 promoter region targeted by ChIP primers, marked in red and used as primers for CHIP assays. Quantitative analysis is presented in the right panel. (*p < 0.05 or **p < 0.01 vs. NG; two‐way ANOVA, *n* = 3 per group).

To investigate whether OPA1 mediates IL‐15’s protective effects on mitochondrial dynamics in podocytes, we performed OPA1 knockdown using OPA1‐specific siRNA (siOPA1). Immunoblotting confirmed successful knockdown, with an ~80% reduction in OPA1 expression 48‐h posttransfection compared to podocytes transfected with scrambled control siRNA, which validated the efficiency of OPA1 knockdown for further experiments. Moreover, the expression of Nephrin and COX IV was decreased upon OPA1 knockdown (Figure 6A). Next, we examined mitochondrial morphology in OPA1‐knockdown podocytes. OPA1 knockdown abolished the protective effects of IL‐15 in HG conditions, resulting in fragmented mitochondrial morphology (Figure 6B). It supports the critical role of OPA1 in mediating IL‐15's protective effects. To further assess the functional impact of OPA1 knockdown, we measured the expression of Nephrin and COX IV. Both Nephrin and COX IV levels were reduced in OPA1‐knockdown podocytes treated with HG, even with IL‐15 supplementation. By contrast, DRP1 phosphorylation levels remained unchanged regardless of OPA1 knockdown (Figure 6C). These results indicate that OPA1 is essential for IL‐15 to preserve mitochondrial function and podocyte integrity under HG conditions. Finally, we explored the mechanism through which IL‐15 regulates OPA1 expression. Given previous findings that IL‐15 can induce histone acetylation [19], we hypothesized that IL‐15 might enhance OPA1 gene transcription via histone modification. Using chromatin immunoprecipitation (ChIP) with an anti‐H3ac antibody, we observed significant enrichment of histone H3 acetylation at the OPA1 promoter following IL‐15 treatment, even under HG conditions (Figure 6D). These findings suggest that IL‐15 enhances OPA1 expression through epigenetic regulation by acetylating histone H3 at the OPA1 promoter, thereby promoting transcription. In summary, our results demonstrate that OPA1 is indispensable for IL‐15‐mediated protection against HG‐induced mitochondrial fission in podocytes. IL‐15 upregulates OPA1 expression via histone H3 acetylation at its promoter, enabling the maintenance of a fused mitochondrial network and preserving podocyte function under diabetic conditions.

### 3.7 The Therapeutic Impact of IL‐15 on the Development of DN

**FIGURE 7:**
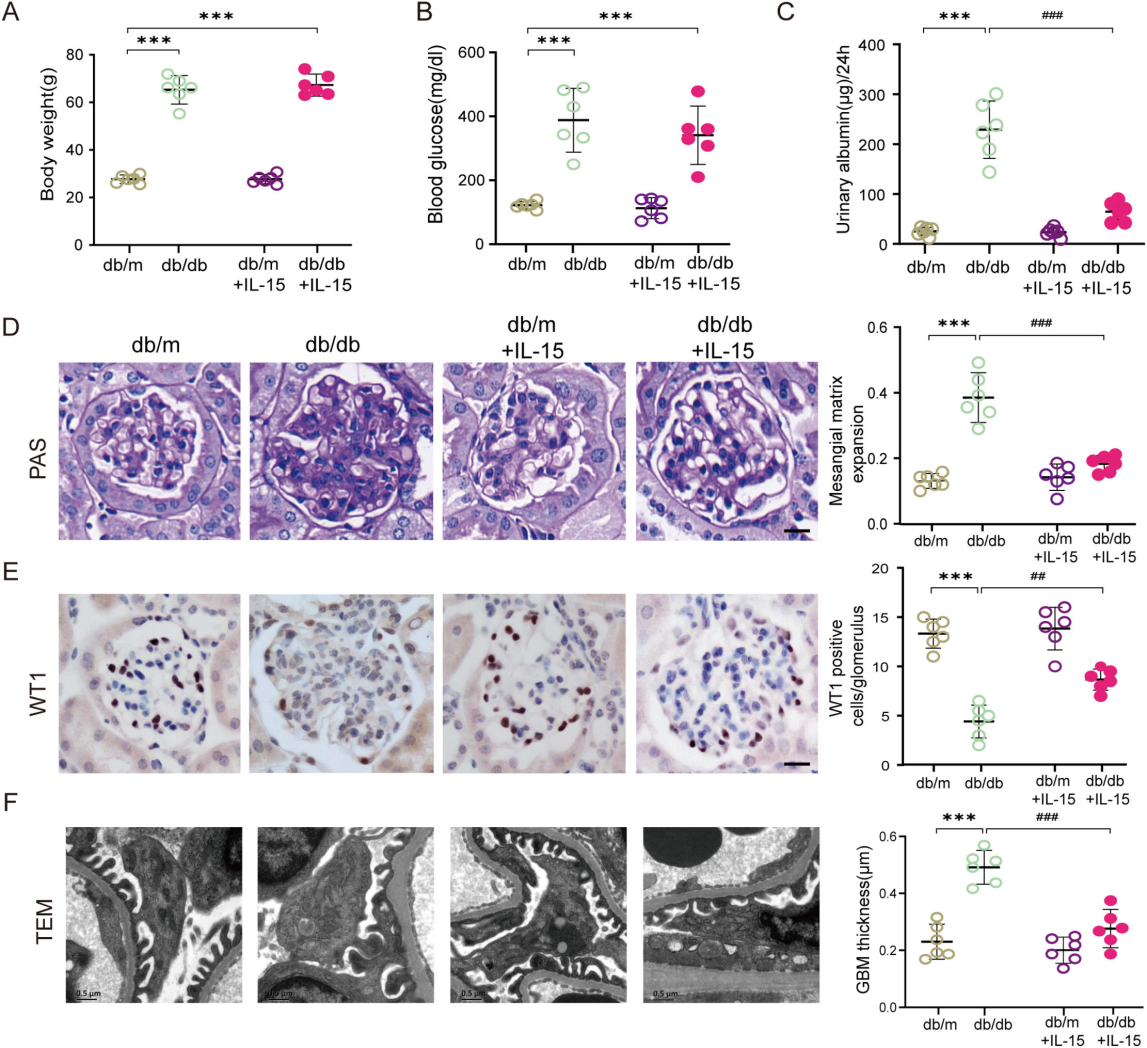
IL‐15 treatment ameliorates DN in mice. (A) Quantification of body weight for mice at 24 weeks of age, grouped by treatment (***p < 0.001 vs. db/m mice; two‐way ANOVA, *n* = 6 per group). (B) Blood glucose levels quantified for mice at 24 weeks of age, grouped by treatment. (***p < 0.001 vs. db/m mice; two‐way ANOVA, *n* = 6 per group). (C) Quantitative analysis of 24‐h urine albumin. (***p < 0.001 vs. db/m mice; ###*p* < 0.001 vs. db/db mice; two‐way ANOVA, *n* = 6 per group). (D) Left panel displays representative images of periodic acid‐Schiff (PAS)‐stained kidney sections. Right panel shows quantification of mesangial matrix. Scale bar represents 20 μm. (***p < 0.001 vs. db/m mice; ###*p* < 0.001 vs. db/db mice; two‐way ANOVA, *n* = 6 per group). (E) Left panel shows representative images of WT1 immunohistochemically stained kidney sections. Right panel presents quantification of the number of WT1 positive cells per glomerulus. Scale bar represents 20 μm. (***p < 0.001 vs. db/m mice; ###*p* < 0.001 vs. db/db mice; two‐way ANOVA, *n* = 6 per group). (F) Left panel shows representative transmission electron micrographs of the glomerular filtration barrier. Scale bar: 500 nm. Right panel presents quantification of the thickness of the glomerular basement membrane. (***p < 0.001 vs. db/m mice; ###*p* < 0.001 vs. db/db mice; two‐way ANOVA, *n* = 6 per group).

To evaluate the therapeutic potential of IL‐15 in DN, we first compared plasma IL‐15 levels between patients with DKD and healthy controls. IL‐15 levels were significantly lower in DKD patients (2.211 ± 0.101 pg/mL) than in healthy controls (3.023 ± 0.138 pg/mL; *p* < 0.0001; Figure S2A). Correlation analysis between plasma IL‐15 levels and urinary albumin‐to‐creatinine ratio (UACR) in 84 diabetic patients revealed that lower IL‐15 levels were associated with more severe albuminuria (*r* = −0.34, *p* < 0.001; Figure S2B). These findings suggest that reduced IL‐15 may contribute to the pathogenesis of DN and provide a rationale for IL‐15 supplementation as a potential therapeutic strategy. To further investigate this, we administered recombinant murine IL‐15 to 5‐month‐old db/db mice intraperitoneally for 4 weeks. Body weight and blood glucose levels were not significantly affected by IL‐15 treatment in either db/m or db/db mice (Figure 7A,B), indicating that IL‐15 does not alter systemic metabolism. Urinary albumin excretion, a key marker of kidney damage in DN, was significantly reduced in IL‐15‐treated db/db mice compared to untreated controls (Figure 7C). Histological examination of kidney sections revealed that IL‐15 markedly reduced mesangial expansion, as quantified by periodic acid‐Schiff (PAS) staining (Figure 7D). Similarly, WT1 staining demonstrated that IL‐15 treatment preserved podocyte numbers in glomeruli, reducing podocyte loss in db/db mice (Figure 7E). Transmission electron microscopy (TEM) further confirmed that IL‐15 mitigated glomerular basement membrane (GBM) thickening in db/db mice (Figure 7F). Collectively, these results indicate that IL‐15 protects against the progression of DN by reducing podocyte injury, improving glomerular structure and decreasing urinary albumin excretion. These findings highlight IL‐15 as a promising targeted therapeutic strategy for mitigating diabetic kidney damage.

## 4 Discussion

Diabetic nephropathy is characterized by early podocyte injury and impaired mitochondrial homeostasis, and accumulating evidence suggests that skeletal muscle‐derived factors may help counteract these pathogenic processes. In this study, we identify IL‐15 as a myokine capable of preserving mitochondrial fusion in podocytes and mitigating high‐glucose‐induced mitochondrial fragmentation. By demonstrating that PGC‐1α activation in skeletal muscle elevates circulating IL‐15 and enhances podocyte mitochondrial fitness through OPA1, our findings provide a reductionist mechanistic framework for muscle–kidney communication in the diabetic setting.

Skeletal muscle expresses numerous exercise‐associated secreted factors [3, 20], including irisin, BDNF and FGF21, several of which have been implicated in renal protection in experimental models [21, 22, 23]. Although these myokines likely act in combination in vivo, our results establish IL‐15 as an additional kidney‐protective mediator whose function had not been previously characterized in DN. Notably, prior work indicates that IL‐15 and irisin act through distinct pathways: Quinn et al. reported that irisin production and secretion are independent of IL‐15, and IL‐15's actions are not mediated by irisin [24], supporting our rationale for investigating IL‐15 as a separate myokine within the muscle–kidney axis.

IL‐15 is secreted primarily by skeletal muscle, macrophages and neutrophils, and its expression increases in response to physical activity [25]. Given that skeletal muscle accounts for nearly 40% of total body mass [26], it likely represents a major source of circulating IL‐15 [27]. Consistent with this, we observed elevated plasma IL‐15 levels in mPGC‐1α mice compared with wild‐type controls. Functionally, IL‐15 is known to improve mitochondrial respiration, suppress lipogenesis and enhance insulin sensitivity through mechanisms linked to increased energy expenditure and weight regulation [17]. In obesity and type 2 diabetes, IL‐15 has been shown to enhance mitochondrial function, regulate lipid and glucose metabolism, counteract TNF‐α–induced metabolic impairment and alleviate endoplasmic reticulum stress [28]. In adipose tissue, IL‐15 increases mitochondrial activity and mass [29], and IL‐15 supplementation has also been reported to improve mitochondrial function in skeletal muscle [30].

Beyond systemic metabolic benefits, IL‐15 exerts renoprotective actions in multiple kidney disease models. In acute kidney injury, IL‐15 reduces tubular epithelial apoptosis, whereas IL‐15Rα deficiency exacerbates renal damage [31]. In CKD, IL‐15 inhibits epithelial–mesenchymal transition by suppressing TGF‐β1 signalling, thereby reducing fibrosis [32]. IL‐15 also possesses anti‐inflammatory and cytoprotective properties mediated through JAK/STAT, PI3K/AKT and MEK/ERK pathways [33]. In autoimmune glomerulonephritis, IL‐15 preserves podocyte morphology and autophagy via STAT5 signalling, reducing FSGS‐like injury [34], and JAK/SYK‐driven IL‐15 signalling provides protection independent of lymphocytes [35]. However, the role of IL‐15 in podocyte pathology during the progression of DN has remained largely unexplored. Our study provides evidence that IL‐15 enhances mitochondrial fitness in diabetic podocytes by promoting OPA1‐dependent mitochondrial fusion.

Mitochondria are highly dynamic organelles that constantly undergo fission and fusion, processes essential for maintaining respiratory capacity, metabolic function and mitochondrial quality control [36]. Disruption of this balance is closely linked to DN progression, with excessive mitochondrial fission recognized as a hallmark of podocyte injury, contributing to increased ROS production and apoptosis [9, 37]. Although prior studies have largely focused on suppressing mitochondrial fission as a therapeutic strategy, our findings highlight enhancing mitochondrial fusion—specifically through OPA1 upregulation—as an effective complementary approach. IL‐15 markedly increased OPA1 expression in podocytes, helping restore a healthier equilibrium between fission and fusion, thereby lowering ROS levels and preserving cellular integrity. Importantly, while excessive fission drives oxidative stress and apoptosis [38], physiological fission is crucial for mitochondrial quality control by segregating damaged mitochondria for removal [39]. Complete inhibition of fission disrupts this process and can worsen mitochondrial dysfunction [40]. By promoting OPA1‐dependent fusion without abolishing fission, IL‐15 provides a balanced means of improving mitochondrial homeostasis while maintaining essential quality control mechanisms.

Several limitations should be acknowledged. First, although our findings focus on podocytes, the effects of IL‐15 on other renal cell types, such as endothelial and mesangial cells, remain to be investigated. Second, although circulating IL‐15 levels were reduced in patients with diabetic kidney disease, the long‐term safety and therapeutic window of IL‐15 administration in diabetes remain uncertain. Third, although IL‐15 clearly promotes OPA1 expression and mitochondrial fusion, the receptor‐proximal mechanisms linking IL‐15Rα activation to OPA1 regulation require further clarification. Finally, our work utilized a reductionist model based on PGC‐1α activation rather than a physiological exercise intervention; integrating formal exercise protocols in future studies will help determine how muscle‐derived IL‐15 contributes to whole‐body metabolic adaptation.

In conclusion, we identify skeletal muscle‐derived IL‐15 as a previously unrecognized mediator of muscle–kidney crosstalk that enhances OPA1‐dependent mitochondrial fusion and promotes podocyte resilience in diabetic nephropathy. These findings expand the mechanistic landscape through which muscle‐derived signals influence renal health and highlight IL‐15 as a potential molecular target to mitigate hyperglycaemia‐induced podocyte injury. Future studies examining how IL‐15 interacts with other myokines, including irisin, will be essential for understanding the integrated endocrine network through which skeletal muscle modulates kidney outcomes.

## Funding

This work was supported by the National Natural Science Foundation of China (81873613 to HP and 82000727 to YL), the Guangdong Provincial Enterprise Joint Fund‐Key projects (2021B1515230005 to HP) and the Basic and Applied Basic Research Foundation of Guangzhou, China (2023A04J1805 to YL). ZYH was supported by the NIH (5R01DK037175).

## Ethics Statement

This study was approved by the Use Committee for animals and the Ethics Committee of the third affiliated Hospital of Sun Yat‐sen University for human plasma. Written consent for participation and publication has been obtained from all the participants in the current study.

## Consent

All authors agree to the publication of this study.

## Conflicts of Interest

The authors declare no competing interests.

## Supporting information


**Table S1:** The sequences of the primers used for PCR were as follows
**Figure S1:** The expression of IL‐15 is increased in mPGC‐1*α* mice.
**Figure S2:** IL‐15R*α* expression in podocytes.
**Figure S3:** Reduced plasma IL‐15 levels with increased albuminuria in DKD patients.

## Data Availability

Data can be accessed by contacting the corresponding authors. scRNA‐seq data are available at GEO with accession number GSE131882.
