# Supplementary material for: IL‐15 Links Muscle–Kidney Crosstalk to Preserving Podocyte Mitochondrial Fusion and Attenuating Diabetic Nephropathy
Source: J Cachexia Sarcopenia Muscle. 2026 Mar 17;17(2):e70256. doi: 10.1002/jcsm.70256 (PMC13140195; doi:10.1002/jcsm.70256)
Supplement: Supplementary file 1 — Table S1: The sequences of the primers used for PCR were as follows Figure S1: The expression of IL‐15 is increased in mPGC‐1α mice. Figure S2: IL‐15Rα expression in podocytes. Figure S3: Reduced plasma IL‐15 levels with increased albuminuria in DKD patients. [file JCSM-17-e70256-s001.docx]

**Supplemental Data**

**IL-15 links muscle-kidney crosstalk to preserve podocyte mitochondrial fusion and attenuate diabetic nephropathy**

Yin Li^1*^, Jialing Rao^1*^, Weiyan Lai^1^, Yuxiang Sun^1^, Hongchun Lin^1^, Jun Zhang^1^, Zengchun Ye^1^, Zhaoyong Hu^2#^, Hui Peng^1#^

^1^Nephrology Division, The Third Affiliated Hospital of Sun Yat-sen University, Guangzhou, China

^2^Nephrology Division, Department of Medicine, Baylor College of Medicine, Houston, TX, USA

*Authors contributed equally to this study.

^#^ Correspondence author Email: [pengh@mail.sysu.edu.cn](mailto:pengh@mail.sysu.edu.cn) or zhaoyonh@bcm. edu

Supplementary Table p.2

Supplementary Figures p.3-6

Supplemental Methods p.7-12

Supplemental references p.12

**Supplementary Table 1.**

**Table S1**. The sequences of the primers used for PCR were as follows:

| Gene | Forward | Reverse |
| --- | --- | --- |
| *Opa1* | CTGTGGGAACGAGTGTCAAC | CTGTCATGAAGCGGGAAAAT |
| *Slc25a4* | TCAATGGGCTGGGCGACTGT | ACCGCTGTCACACTCTGGGCAA |
| *Slc25a12* | GGCAGGAGTAGCTGATCAAACCA | CACCTCTCCATTTCCGCTCTTGT |
| *Timm10b* | TGTGTTTCCAGCGCTGTGTG | GCGAGTCTCTGGTCTGTTCTGCA |
| *Cox10* | AGGTGCCGTTCGACTCAAACA | CCACGGCTCCAACCCAGGTATT |
| *Bdnf* | GCCCAACGAAGAAAACCATA | GCTGTGACCCACTCGCTAAT |
| *Ctsb* | TCCTTCTTTCTTGCCTGCTG | GTGCCACACAGCTTCTTCAG |
| *Ctsd* | CAACAGAAGCTGGTGGACAA | TAGGCCTTTCGAGTGACGTT |
| *Fgf-21* | CTGGGGGTCTACCAAGCATA | CACCCAGGATTTGAATGACC |
| *Igf-1* | GAAAATCAGCAGCCTTCCAA | GTCTCTGGTCCAGCTGTTGGT |
| *Il-15* | TGAGGAATACATCCATCTCGTG | TGGCCTCTGTTTTAGGGAGA |
| *Pedf* | CCTGTGTGCTACTCCTCTGGA | GACCTTGAAGAAGGGGTCCT |
| *β-actin* | TCGTACCACAGGCATTGTGATGGA | TGATGTCACGCACGATTTCCCTCT |

**Supplementary Figure 1.**


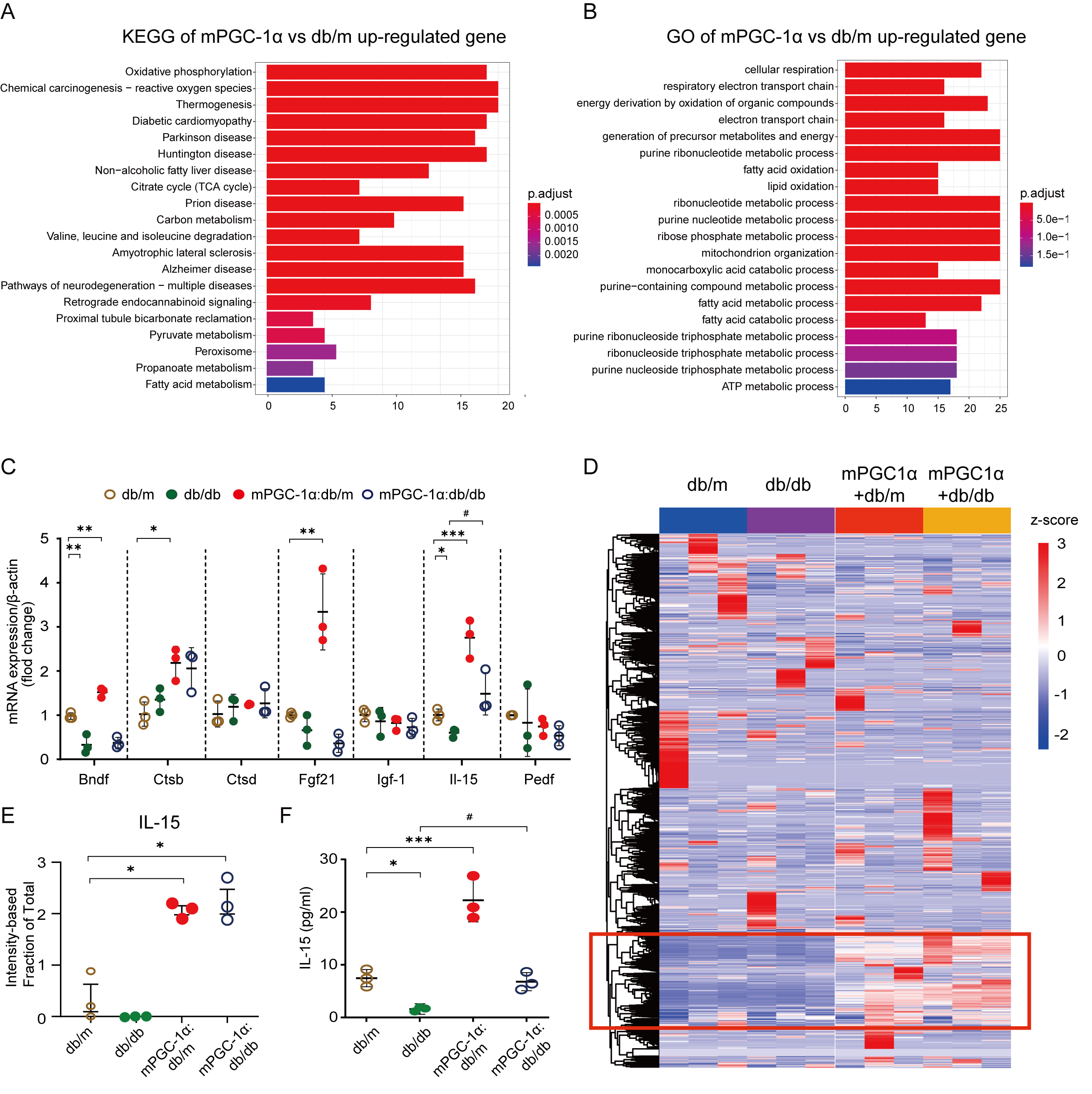


**Fig.S1. The expression of IL-15 is increased in mPGC-1α mice.**

1. Kyoto Encyclopedia of Genes and Genomes (KEGG) functional enrichment of up-regulated expressed genes for muscles from mPGC-1α:db/m vs db/m.
2. Gene Ontology (GO) functional classification of up-regulated expressed genes for muscles from mPGC-1α:db/m vs db/m.
3. Real-time PCR analysis of the expression of seven known myokines related to mitochondrial function in skeletal muscles from four groups of mice: db/m, db/db, mPGC-1α:db/m, and mPGC-1α:db/db. (**P*<0.05 or ***P*<0.01 or ****P*<0.001 vs. db/m; #*P*<0.05 vs. db/db; two-way ANOVA, n=3 per group).
4. Heat map showing the expressions of protein in muscles from four different groups of mice: db/m, db/db, PGC-1α: db/m, PGC-1α: db/db. The red box marks the consistently upregulated protein in mPGC-1α mice with or without diabetes.
5. Bar graph displays proteomic analysis of IL-15 expression in skeletal muscles from four groups of mice above (**P*<0.05 vs. db/m; two-way ANOVA, n=3 per group).
6. Bar graph displays plasma concentrations of IL-15 in each mouse group, quantified by ELISA. (**P*<0.05 or ****P*<0.001 vs. db/m; #*P*<0.05 vs. db/db; two-way ANOVA, n=3 per group).

**Supplementary Figure 2.**


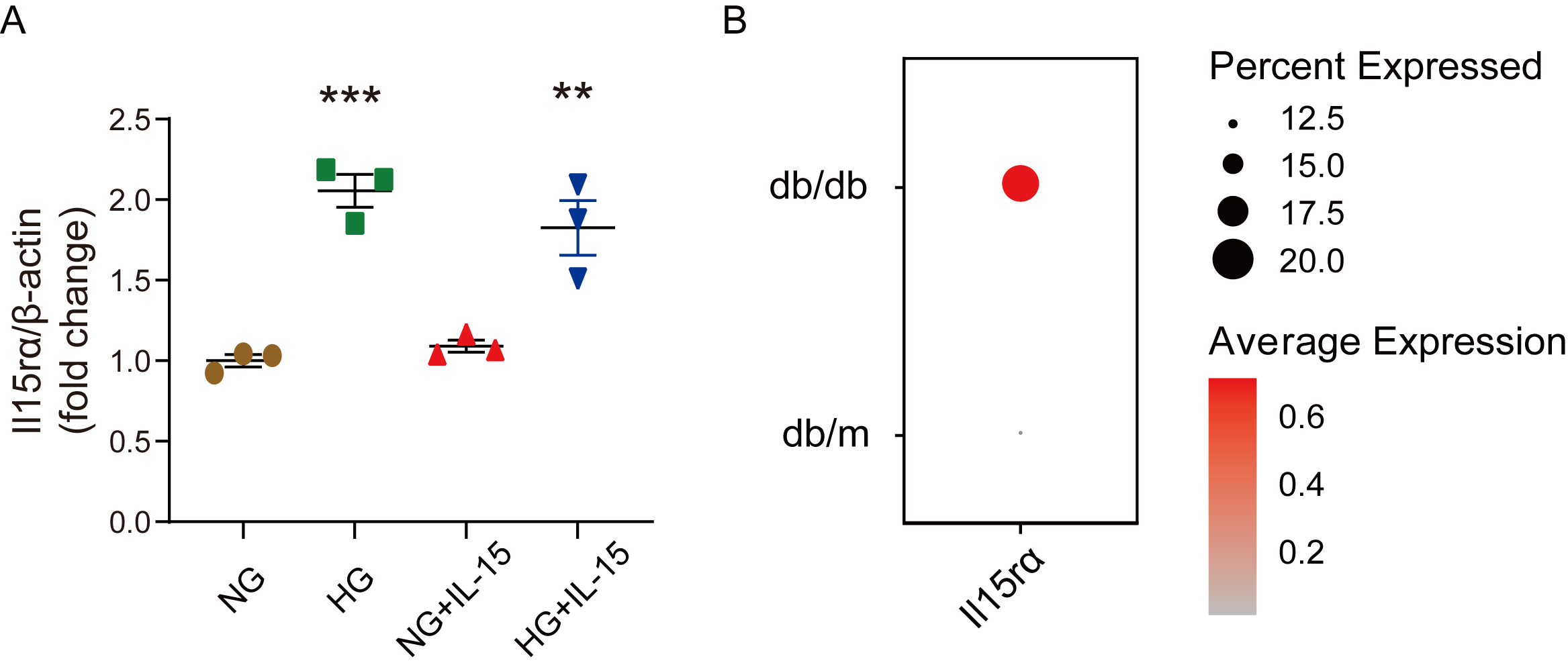


**Figure S2. IL-15Rα expression in podocytes.**

1. Expression of Il-15rα in cultured podocytes under the indicated conditions. Podocytes were treated with normal glucose (NG), high glucose (HG), NG + IL-15, or HG + IL-15. (***P*<0.01 or ****P*<0.001 vs. NG; two-way ANOVA, n=3 per group).

B. Single-cell RNA-seq analysis of Il-15rα expression in podocytes from diabetic (db/db) and control (db/m) mice using dataset GSE184652. Dot plot shows the percentage of Il-15rα-expressing podocytes (dot size) and average expression level (color scale).


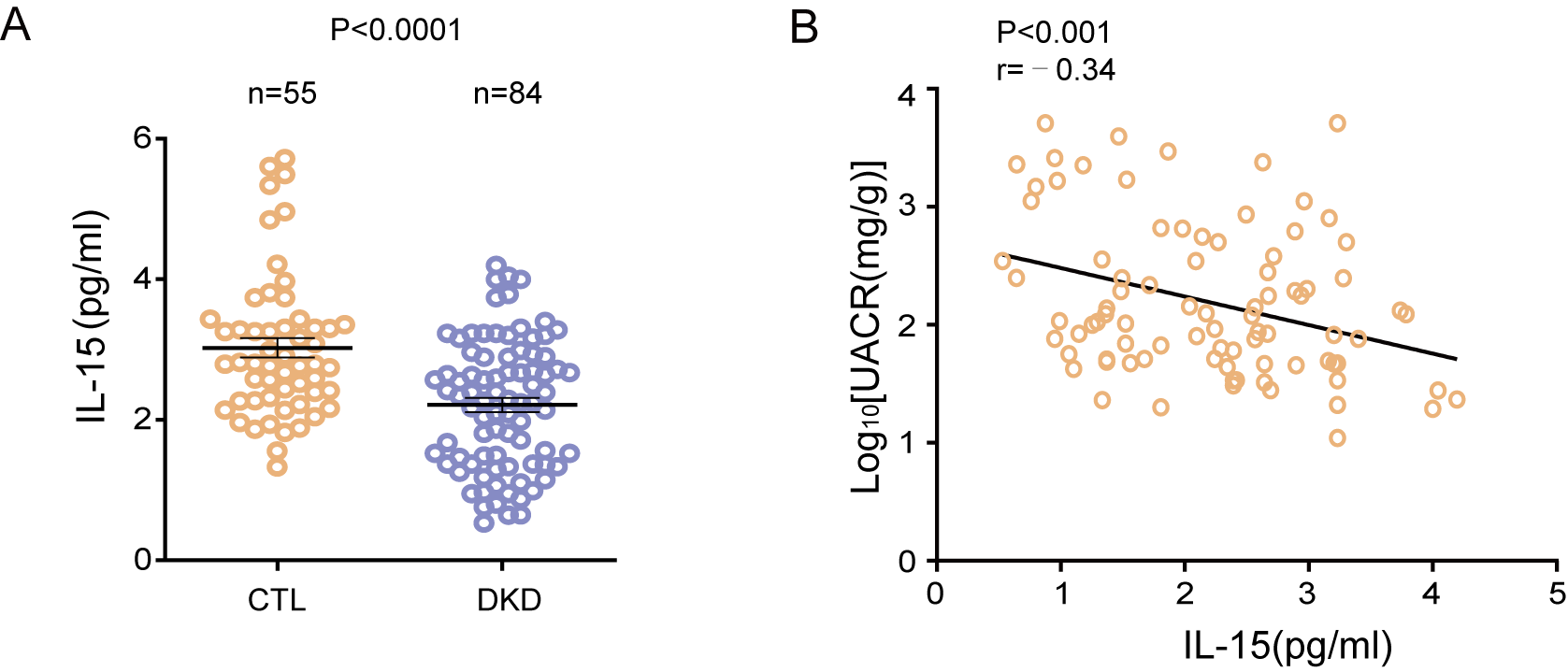


**Fig.S3. Reduced plasma IL-15 levels with increased albuminuria in DKD patients.**

1. Scatter plots illustrate the plasma IL-15 concentrations in patients with diabetic kidney disease (DKD; n=84) compared to control individuals (CTL; n=55).
2. Analysis of the correlation between plasma IL-15 levels and urinary albumin-to-creatinine ratio (UACR) in patients with diabetic kidney disease (DKD; n=84).

**Supplemental Methods**

**Urinary albumin and podocyte detection**

Individual mice were housed in metabolic cages for 24-hour urine collection with free access to food and water. Urine samples were centrifuged at 1,800 rpm for 15 minutes at 4°C, and the supernatant was aliquoted and stored at -80°C until analysis.

Urinary albumin detection: Aliquots of urine samples were mixed with 6X SDS loading buffer, separated on 4%-20% gradient SDS-PAGE gels, and stained with Coomassie blue. Albumin bands (~65 kDa) were quantified using ImageJ software, with bovine serum albumin standards for calibration.

Podocyte detection in urine: After removing the supernatant, the pellet was resuspended in 2X SDS loading buffer and separated on 4%-20% SDS-PAGE gels. Proteins were transferred to nitrocellulose membranes, incubated with an anti-podocin antibody (Santa Cruz), followed by HRP-conjugated secondary antibody incubation, and detected using enhanced chemiluminescence (ECL).

**Histology and immunohistochemistry**

Kidneys were fixed in 10% neutral-buffered formalin, embedded in paraffin, and sectioned at 4 µm thickness. For Periodic Acid-Schiff (PAS) staining, sections were deparaffinized, rehydrated, and stained with PAS (Sigma) following the manufacturer’s guidelines. Mesangial matrix expansion was quantified from five randomly selected glomeruli per section using Adobe Photoshop, with a minimum of six mice per group analyzed. For WT1 immunostaining: sections underwent heat-induced antigen retrieval in 10 mM sodium citrate buffer (pH 6.0) and were treated with 0.3% hydrogen peroxide for 20 minutes to quench endogenous peroxidase activity. After blocking in 5% bovine serum albumin for 30 minutes, sections were incubated overnight at 4°C with an anti-WT1 antibody (Santa Cruz). The following day, sections were washed and incubated with a biotinylated secondary anti-rabbit antibody (Vector Laboratories) for 30 minutes at room temperature. Detection was performed using a Vectastain ABC Kit (Vector Laboratories), and slides were counterstained with hematoxylin. Podocyte numbers were counted from 20 randomly selected glomeruli per section.

**Immunofluorescence**

Frozen kidney tissues were cryosectioned at 5 μm thickness and fixed in cold acetone for 10 minutes. Sections were blocked with 5% bovine serum albumin (BSA) for 30 minutes at room temperature to reduce nonspecific binding. Primary antibodies, including anti-IL-15Rα and anti-WT1 (Santa Cruz Biotechnology), were diluted in 5% BSA and incubated overnight at 4°C. After washing with PBS, sections were incubated with Alexa Fluor–conjugated secondary antibodies (Life Technologies) for 1 hour at room temperature in the dark. Nuclei were counterstained with DAPI (Thermo Fisher Scientific). Fluorescence images were acquired using a Nikon Eclipse Ti confocal microscope and processed using ImageJ (NIH).

**Western blot**

Kidney tissue was homogenized in RIPA buffer (GBiosciences) supplemented with protease and phosphatase inhibitors (Thermo Fisher Scientific). Lysates were incubated on ice for 15 minutes and centrifuged at 12,000 rpm for 15 minutes at 4°C. Protein concentrations were measured using the BCA assay (Thermo Fisher Scientific). Equal amounts of protein were mixed with 2X SDS loading buffer, separated on 4%-20% gradient SDS-PAGE gels, and transferred to nitrocellulose membranes. Membranes were blocked with 5% non-fat dry milk in TBST for 1 hour at room temperature. The following primary antibodies were used: Nephrin (R&D Systems); OPA1, COX IV, Drp1, p-Drp1(Ser616) (Cell Signaling Technology); Podocin, IL15Rα (Santa Cruz Biotechnology) and β-actin (Sigma-Aldrich). After overnight incubation at 4°C, membranes were washed and incubated with HRP-conjugated secondary antibodies for 1 hour at room temperature. Protein bands were visualized using an ECL detection system (Bio-Rad laboratories), and band intensities were quantified using ImageJ software. Three independent biological replicates were analyzed.

**PCR and PCR Array**

Total RNA was extracted from podocytes and mouse muscle tissues using QIAzol Lysis Reagent (QIAGEN) and quantified using a NanoDrop spectrophotometer (Thermo Fisher Scientific). cDNA was synthesized using qScript cDNA SuperMix (Quanta Biosciences). Quantitative PCR (qPCR) was performed using SYBR Green FastMix (Quanta Biosciences) on a CFX96 Real-Time PCR System (Bio-Rad), and results were normalized to β-actin as a housekeeping gene.

For the PCR array, the Mouse Mitochondria RT2 Profiler PCR Array (QIAGEN) was used to evaluate mitochondrial-related gene expression. RNA was reverse-transcribed using the RT2 First Strand Kit (QIAGEN), and the PCR array was performed according to the manufacturer’s instructions. Data analysis was conducted using the online RT² Profiler PCR Array Data Analysis software (QIAGEN). The sequences of the primers used for PCR were shown in Table S1.

**Transmission electron microscopy (TEM)**

Cortical kidney tissue fragments were fixed in 2.5% glutaraldehyde in 0.1 M cacodylate buffer at 4°C for 2 hours. Samples were post-fixed in 1% osmium tetroxide for 1 hour, dehydrated through a graded ethanol series, and embedded in Epon resin. Ultrathin sections (70 nm) were cut using an ultramicrotome (Leica UC7) and stained with uranyl acetate and lead citrate. Sections were imaged using a JEM-1400 transmission electron microscope (JEOL) at 80 kV. Images were analyzed using ImageJ software to evaluate podocyte morphology and glomerular basement membrane thickness. A minimum of 10 glomeruli per mouse (6 mice per group) were assessed.

**Mitochondrial function**

Live podocytes were stained with MitoTracker Red CMXRos (300 nM; Invitrogen) for 15 minutes at 37°C to evaluate mitochondrial morphology. The mitochondrial size measurements were obtained using ImageJ with the mitochondrial analyzer plugin as previously described [S1].

Mitochondrial ROS (MtROS) production was assessed using MitoSOX Red (5 μM; Invitrogen) for 10 minutes at 37°C. After staining, cells were washed with PBS and mounted in the antifade medium. Images were acquired using a Nikon Eclipse Ti confocal microscope with 40x and 100x objectives. Integrated fluorescence intensity was analyzed using ImageJ software, with at least 100 cells evaluated per condition across three independent experiments.

**Cell Transfections**

For gene silencing, podocytes were transfected with OPA1-specific siRNA (Sigma) using Lipofectamine RNAiMAX (Invitrogen) according to the manufacturer's protocol. Knockdown efficiency was verified by Western blotting. For gene overexpression, podocytes were infected with adenovirus at a defined multiplicity of infection (MOI), using the formula: virus volume (mL) = (MOI × number of cells) / virus titer (PFU/mL). In siRNA experiments, cells were maintained for 48 hours post-transfection before HG/NG ± IL-15 treatment for an additional 4 days. For adenoviral infection, cells were incubated with virus for 24–48 hours before subsequent treatment.

**Chromatin immunoprecipitation assay (ChIP)**

A Chromatin Immunoprecipitation (ChIP) assay was performed to assess acetyl-histone modifications at the OPA1 promoter following previous described protocol [S2] and the manufacturer’s instruction (Merck-Millipore, #17-245). Briefly, podocytes were treated with recombinant mouse IL-15 (1 ng/ml) or vehicle control for 24 hours. Cross-linking was achieved using 1% formaldehyde for 10 minutes at room temperature, followed by quenching with glycine. Chromatin was extracted, sheared to an average fragment size of 200-500 bp using sonication, and immunoprecipitated overnight at 4°C using anti-acetyl-histone H3 antibodies (Merck-Millipore, #06-599).

The immunoprecipitated chromatin was subjected to PCR amplification using OPA1 promoter-specific primers: sense, TTCCACGCCTTTAGCCCTTC; antisense, GCCCTCTTGCTTCCGATTTG. The exact primer locations are indicated in Figure 5H. PCR products were resolved via electrophoresis on a 2% denaturing polyacrylamide gel, visualized using ethidium bromide staining, and imaged with a Bio-Rad Gel Doc system. One percent of the total chromatin input from each sample was reserved as a control for normalization. The ChIP assay was repeated three times for consistency.

**Measurement of plasma IL-15 concentrations**

Plasma samples from 84 patients with diabetic kidney disease (DKD) and 55 healthy volunteers were collected under protocols approved by the Ethics Committee of the Third Affiliated Hospital of Sun Yat-sen University. Written informed consent was obtained from all participants. Plasma was collected in EDTA tubes, centrifuged at 1,500 g for 15 minutes at 4°C, and stored at -80°C. IL-15 concentrations were measured using an electrochemiluminescence assay (MSD K151URK-1 kit) according to the manufacturer’s protocol. Samples were analyzed in duplicate, and inter-assay and intra-assay coefficients of variation were <10%.

**Bulk RNA sequencing analysis**

Bulk RNA was extracted from the muscle of db/m, db/db, mPGC-1α:db/m and mPGC-1α:db/db mice. We prepared Sequencing libraries by using the Illumina TruSeq protocol and sequenced on an Illumina NovaSeq 6000 platform (paired-end 2x150 base pair) to an average depth of 43.5 million reads per sample, and mapped reads to the mouse genome (UCSC mm10) using HISAT2 (v.2.1.0). HTSeq-count was used to quantify gene expression with the mm10 gene annotation GTF file from the UCSC genome browser. We performed differential expression analysis through the edgeR package (version 3.28.1). Further analyses, such as principal component analysis (PCA), and enrichment analysis, were conducted by applying FactoMineR (version 2.4) and clusterProfiler (version 4.13.4), respectively.

**Muscle peptide preparation and LC-MS/MS analysis**

TA muscle samples (~1 mg wet weight) from each mouse were homogenized in a buffer containing 8 M urea, and protein concentration was determined by BCA assay. An aliquot equivalent to 500 µg of total protein per sample was reduced with 10 mM DTT, alkylated with 20 mM iodoacetamide, and digested overnight with sequencing-grade trypsin. The resulting peptides were desalted using C18 StageTips, vacuum dried, and resuspended in 2% acetonitrile with 0.1% formic acid . Approximately 1 µg of peptides per sample was injected for analysis on an UltiMate™ 3000 RSLCnano system coupled to a Fusion™ Tribrid™ mass spectrometer (Thermo Scientific™). Peptides were separated on a C18 reversed-phase column (75 µm ID × 25 cm, 1.9 µm particles) using a 60-minute linear gradient from 4% to 32% ACN in 0.1% FA at a flow rate of 300 nL/min. The mass spectrometer operated in Data-Dependent Acquisition (DDA) mode, with MS1 scans acquired in the Orbitrap at 120,000 resolution (375–1500 m/z). The most intense precursors were selected for Higher-energy Collisional Dissociation (HCD) fragmentation and MS2 detection in the Orbitrap at 30,000 resolution, with dynamic exclusion enabled for 30 seconds. The resulting raw data files were processed and analyzed using Thermo Scientific Proteome Discoverer (version 2.1). Protein identification was performed using the Sequest HT search engine against the UniProt mouse reference proteome. A false discovery rate (FDR) of 1% was applied at both the peptide and protein levels. Label-free quantification was calculated based on precursor ion abundance.

**Supplemental references**

S1. Chen Y, Zhang J, Zhang M et al (2021) Baicalein resensitizes tamoxifen-resistant breast cancer cells by reducing aerobic glycolysis and reversing mitochondrial dysfunction via inhibition of hypoxia-inducible factor-1alpha. Clin Transl Med 11:e577. https://doi.org/10.1002/ctm2.577

S2. Musikacharoen T, Yoshikai Y, Matsuguchi T (2003) Histone acetylation and activation of camp-response element-binding protein regulate transcriptional activation of mkp-m in lipopolysaccharide-stimulated macrophages. J Biol Chem 278:9167-9175. https://doi.org/10.1074/jbc.M211829200
